# Correction to: Extracellular Vesicles: Biomarkers, Therapeutics, and Vehicles in the Visual System

**DOI:** 10.1007/s40135-018-0156-5

**Published:** 2018-01-23

**Authors:** Yolandi van der Merwe, Michael B. Steketee

**Affiliations:** 1 Department of Bioengineering, University of Pittsburgh, Pittsburgh, PA 15260 USA; 2 Department of Ophthalmology, University of Pittsburgh, Pittsburgh, PA 15216 USA; 3 McGowan Institute for Regenerative Medicine, University of Pittsburgh, 450 Technology Drive, Suite 300, Pittsburgh, PA 15219 USA; 4 Center for Neuroscience, University of Pittsburgh, Pittsburgh, PA 15261 USA

**Correction to: Curr Ophthalmol Rep (2017) 5:276–282**

10.1007/s40135-017-0153-0

The article Extracellular Vesicles: Biomarkers, Therapeutics, and Vehicles in the Visual System, written by Yolandi van der Merwe and Michael B. Steketee, was originally published Online First without open access. After publication in volume [5], issue [4], page [276–282] the author decided to opt for Open Choice and to make the article an open access publication. Therefore, the copyright of the article has been changed to © The Author(s) 2018 and the article is forthwith distributed under the terms of the Creative Commons Attribution 4.0 International License (http://creativecommons.org/licenses/by/4.0/), which permits use, duplication, adaptation, distribution and reproduction in any medium or format, as long as you give appropriate credit to the original author(s) and the source, provide a link to the Creative Commons license, and indicate if changes were made.

